# Depression and anxiety in patients with and without same-sex attraction: differences in clinical expression, lifestyle factors, and vulnerability indicators

**DOI:** 10.1002/brb3.363

**Published:** 2015-07-14

**Authors:** Henny M W Bos, Lynn Boschloo, Robert A Schoevers, Theo G M Sandfort

**Affiliations:** 1 Faculty of Social and Behavioral Sciences, Research Institute of Child Development and EducationNieuwe Prinsengracht 130, 1018 VZ, Amsterdam, the Netherlands; 2 Faculty of Medical Sciences, Academic Centre of Psychiatry, University of GroningenHanzeplein 1, 9713 GZ, Groningen, the Netherlands; 3 Division of Gender, Sexuality and Health, New York Psychiatric Institute and Department of Psychiatry, Columbia University1051 Riverside Drive, Unit 15, New York, New York, 10032

**Keywords:** Anxiety disorders, clinical expression, depressive disorders, risk factors, same-sex attraction

## Abstract

**Background:**

The aim of this study was to compare clinical expressions (severity and loneliness), lifestyle factors (substance use), and vulnerability indicators (stressful childhood experiences) in patients with any same-sex attraction versus heterosexual patients diagnosed with depression and/or anxiety disorder. Little is known about this, even though it is now well documented that depression and anxiety are more prevalent among persons with same-sex attraction.

**Method:**

Data, derived from the Netherlands Study of Depression and Anxiety (NESDA), allowed us to compare patients with a same-sex (*n *=* *122) and an exclusively opposite-sex (*n *=* *1658) attraction. Persons with same-sex attraction included persons who were attracted to both sexes. Data were collected by means of the Composite International Diagnostic Interview and paper-and pencil questionnaires.

**Results:**

Seven percent of the patients reported any same-sex orientation. Clinical expression of depression and anxiety did not differ in relation to sexual attraction. Regarding substance use, same-sex attracted women reported more drug use than heterosexual women (drug use: 16.2% vs. 6.6%, *P *=* *0.003). Regarding stressful childhood experiences, men with any same-sex attraction reported more sexual abuse during childhood than men with a heterosexual orientation (20.4% vs. 8.5%, *P *=* *0.005).

**Conclusions:**

For women with same-sex attraction substance use (especially illicit drug use) might be a coping mechanism to deal with existing symptoms or with the minority stressors they have to deal with; for same-sex attracted men stressful childhood experiences might reflect an aspect of etiology.

## Introduction

Several studies with large probability samples have shown that persons with a same-sex sexual orientation have a greater risk of developing psychopathology than heterosexual people, and the health disparities revealed to be larger for bisexual than for gay and lesbian people (Cochran and Mays 2000; Sandfort et al. 2001; Bostwick et al. 2010; Gevonden et al. 2014; Elliott et al. 2015). These findings have been found independently of whether sexual orientation was assessed as behavior, identity, or attraction. These sexual orientation-related mental health disparities have not only been found on a lifetime basis but also when assessed for shorter time periods, such as the past year (King et al. 2008). Furthermore, these studies with large probability samples also showed that unhealthy lifestyle factors such as substance use (Cochran et al. 2004) and vulnerability factors such as stressful childhood experiences (Schneeberger et al. 2014) are more prevalent in sexual minorities.

An explanation for these health disparities is offered by the sexual minority stress model. This model identifies several types of minority stress, including victimization during childhood and adulthood (Meyer 2003). Several studies of sexual minorities showed that experiences of rejection because of sexual orientation was related to mental health problems such as depression and suicidality (De Graaf et al. 2006; Zietsch et al. 2012). From the perspective of the minority stress model, a higher prevalence of substance use might be seen as a coping mechanism to deal with the minority stressors lesbian, gay, and bisexual (LGB) people experience (Meyer 2003). It has also been shown that individuals who as an adult identify as lesbian, gay, or bisexual (LGB) may during their childhood display behavior that causes them to be singled out and bullied by peers, or misunderstood and maltreated by their parents (Gevonden et al. 2014).

Although studies have found that sexual minorities have a greater risk of developing psychopathology than heterosexual persons, there are only a few studies that compared sexual minorities and heterosexual persons diagnosed with a mental health disorder. Studies on participants with a diagnosis primarily focused on borderline disorders (Zubenko et al. 1987; Dulit et al. 1993; Grant and Potenza 2006; Bradford et al. 2008). It was found, for example, that patients with a borderline personality disorder were significantly more likely than patients with other personality disorders to report a homosexual or bisexual orientation or intimate same-sex relationships. To our knowledge, no studies have investigated differences between people with a same-sex attraction and heterosexual persons diagnosed with depressive and/or anxiety disorders.

## Aim of the Study

The aim of the present study is to investigate whether there are sexual orientation-related differences in clinical expression (severity of the disorders and loneliness), lifestyle factors (substance use), and vulnerability factors (stressful childhood experiences) on a sample of patients diagnosed with a depressive and anxiety disorders. In the present study we explored these questions using data from the Netherlands Study of Depression and Anxiety (NESDA), which is an ongoing cohort study aimed at examining the long-term course and consequences of depressive and anxiety disorders in the adult (18–65 years) population (Penninx et al. 2008, 2011). The survey instrument used in NESDA includes a question about sexual attraction, which makes it possible to examine whether sexual attraction is related to clinical expression, lifestyle factors and vulnerability indicators. We focused on patients who were all diagnosed with a past-year DSM-IV depressive and/or anxiety disorder, and compared persons who report any same-sex attraction to persons who are heterosexual.

Studies have found that cumulative exposure to stress-related societal discrimination can have deleterious effects on mental and behavioral health of sexual minority populations (Bloomfield et al. 2011). Therefore, we expect that in a sample of patients with a depressive and/or anxiety disorder we will find significant differences between those with same-sex attraction and opposite-sex attraction, such as higher scores on severity of the disorders and loneliness (clinical expression), and higher prevalence of substance use (lifestyle factor), and stressful childhood experiences (vulnerability factors). Understanding potential differences on these variables might improve clinical treatment of disorders in patients with a same-sex attraction.

## Materials and Methods

### Sample

The sample for the present analysis consists of persons who were diagnosed with a past-year depressive and/or anxiety disorder and who also answered the question about reported sexual attraction (*n *=* *1780). These persons were part of NESDA, an ongoing cohort study aimed at examining the long-term course and consequences of depressive and anxiety disorders in the adult (18–65 years) population. A total of 2981 participants were included at the baseline assessment in 2004–2007, consisting of patients with a past-year (60%) or prior history (18%) of depressive and/or anxiety disorder as well as healthy controls (22%) (Penninx et al. 2011). To represent various settings and stages of psychopathology, participants were recruited from the community (19%), primary care (54%), and outpatient mental health care services (27%). Community-based participants had previously been identified in a population-based study; primary care participants were identified through a three-stage screening procedure (involving the Kessler-10 (Kessler et al. 2002) and the short-form Composite International Diagnostic Interview (Kessler et al. 1998) (CIDI) by phone) conducted among a random sample of patients of 65 General Practitioners; and mental health care participants were recruited consecutively when newly enrolled at one of the participating mental health organization locations. Participants with insufficient command of the Dutch language or a primary clinical diagnosis of bipolar disorder, obsessive compulsive disorder, severe substance use disorder, psychotic disorder or organic psychiatric disorder, as reported by them or their (mental) health practitioner, were excluded. Of the 1783 participants diagnosed with a past-year depressive and/or anxiety disorder at NESDA baseline assessment, three did not report information about their sexual attraction and were excluded for the current analysis, resulting in an analytic sample of 1783 (60% of the 2981). A detailed description of the NESDA study design and sampling procedures can be found elsewhere (Penninx et al. 2008, 2011). The research protocol for NESDA was approved by the Ethical Committee of participating universities and all participants provided written informed consent.

### Instruments

The assessment consisted of an extended face-to-face interview, including a standardized diagnostic psychiatric interview, and paper-and-pencil questionnaires.

#### Sexual attraction

Sexual attraction was operationalized based on the question: “Do you prefer to have sex with men, women, or both?” which in the Dutch translation is an indication of sexual attraction. We categorized men as having any same-sex attraction if they answered this question with “men” and “men and women”. Women were categorized as having any same-sex attraction when their answers were “women” and “men and women”. Other persons were categorized as having exclusive heterosexual attraction.

#### Clinical expression

Severity of depressive symptoms was defined as the total score on the 30-item self-report Inventory of Depressive Symptoms (IDS) (Rush et al. 1996), whereas severity of anxiety symptoms was defined as the total score on the 21-item self-report Beck Anxiety Inventory (BAI) (Beck et al. 1988). Emotional and social loneliness were assessed with the 11-item De Jong Gierveld self-report questionnaire (De Jong Gierveld and Kamphuis 1985; De Jong Gierveld and Van Tilburg 2010).

#### Lifestyle factors: Substance use

Substance use included alcohol use, illicit drug use, and smoking (Penninx et al. 2008; Boschloo et al. 2014). Alcohol use, defined as the average number of drinks per day, was based on two items concerning the average frequency of drinking and the amount of drinks on a typical drinking day in the past year. Illicit drug use (cannabis, ecstasy, speed, cocaine, heroin or LSD) in the past month was assessed with a self-report questionnaire. Information about current smoking status was obtained during the interview.

#### Vulnerability indicators: Stressful childhood experiences

Stressful childhood experiences were based on the Childhood Trauma Inventory (Wiersma et al. 2009), which distinguishes emotional neglect, psychological neglect, physical abuse, and sexual abuse before the age of 16 years.

### Statistical analyses

Analyses were conducted using SPSS version 20 statistical software (SPSS Inc, Chicago, IL) and were carried out for men and women separately. Chi-square tests and independent *t*-tests were used for the analyses of categorical and continuous variables, respectively. For the comparisons on the clinical expression variables (severity of the disorders and loneliness), lifestyle factors (substance use), and vulnerability indicators (stressful childhood experiences) between patients with and without same-sex attraction a Bonferonni correction was calculated to reduce the chance of a Type I error due to multiple testing. Based on this correction, differences with a *P* < 0.01 were considered significant. To assess whether associations with sexual attraction were confounded by demographic characteristics we also conducted multivariable logistic regression analyses as an additional sensitivity analyses.

## Results

The proportion of patients with any same-sex attraction was 9.3% (*n *=* *54) for men and 5.7% (*n *=* *68) for women. The numbers of men and women who reported sexual attraction to “men and women” were respectively 10 and 43. Because these subsamples of either exclusively gay or bisexual people are too small to analyze separately we combined them as having any same-sex attraction. Same-sex attraction was significantly more often reported by men than by women (*χ*^2^ = 7.96, df = 1, *P *=* *0.005).

Sample demographic characteristics are presented in Table 2005. Compared to men with heterosexual attraction, same-sex attracted men were more likely to be single, and also had finished their education at a higher level. There was no significant difference in age. The only sexual attraction-related difference for women was related to education: women with same-sex attraction had completed education at a higher level than women with heterosexual attraction.

**Table 1:** Demographic characteristics by sexual attraction, separately for men and women

	Men		Women	
	Heterosexual attraction (*n *=* *528)	Any same-sex attraction (*n *=* *54)	Heterosexual attraction (*n *=* *1130)	Any same-sex attraction (*n *=* *68)
Age in years				
M	43.2	44.9	40.3	39.6
SD	11.6	10.6	12.6	12.9
	*t *=* *−1.01, *P *=* *0.312		*t *=* *0.42, *P *=* *0.677	
Education level, %				
Basic^1^	09.1	01.9	08.8	01.5
Intermediate^2^	64.6	4.41	62.5	41.2
High^3^	26.3	53.7	28.8	57.4
	*χ*^2^ = 18.94, *P *<* *0.001		*χ*^2^ = 26.09, *P *<* *0.001	
Relationship status, %				
No partner	32.4	53.7	34.2	39.7
Partner	67.6	46.3	65.8	60.3
	*χ*^2^ = 9.87, *P *=* *0.002		*χ*^2^ = 0.87, *P *=* *0.350	

1 Participants who did not finished or only finished elementary education.

2 Participants who only completed: (1) lower vocational; (2) general intermediate, intermediate vocational or (3) general secondary education.

3 Participants who completed: (1) higher vocational; (2) college of (3) university education.

M, Mean; SD, standard deviation.

### Clinical expression

As shown in Table 1988, there were no significant differences regarding the severity of the depressive and/or anxiety disorders between male patients with any same-sex attraction and those with heterosexual attraction. Differences in emotional loneliness and social loneliness were also not significant. The results were exactly the same for women: there were no significant differences between female patients with and without same-sex attraction in severity of disorders and emotional and social loneliness.

**Table 2:** Clinical expression, lifestyle factors, and vulnerability indicators for patients with and without same-sex attraction, separately for men and women^1^

	Men				Women			
	Heterosexual attraction (*n* = 528)	Any same-sex attraction (*n* = 54)	*χ*^2^/*t*	*P*	Heterosexual attraction (*n* = 1130)	Any same-sex attraction (*n* = 68)	*χ*^2^/*t*	*P*
	Mean (SD) or % (*n*)	Mean (SD) or % (*n*)			Mean (SD) or % (*n*)	Mean (SD) or % (*n*)		
Clinical expression								
Severity of disorder								
Depressive symptoms (min = 0, max = 84)	29.0 (12.9)	27.9 (12.5)	0.57	0.567	28.7 (12.2)	26.4 (11.0)	1.52	0.130
Anxiety symptoms (min = 0, max = 63)	16.4 (10.7)	14.8 (10.7)	1.03	0.304	17.1 (10.8)	14.9 (07.9)	2.16	0.034
Loneliness								
Emotional loneliness (min = 0, max = 5)	03.2 (01.9)	03.7 (02.1)	−1.82	0.069	02.9 (02.1)	02.6 (02.2)	1.26	0.209
Social loneliness (min = 0, max = 6)	03.0 (01.6)	02.8 (01.9)	1.06	0.358	02.6 (01.7)	02.8 (01.8)	1.47	0.143
Lifestyle factors								
Substance use								
Alcohol use in drinks/day	1.4 (1.9)	1.6 (1.9)	−0.44	0.661	0.7 (1.2)	1.2 (1.6)	−2.24	0.028
Illicit drug use (yes)	11.7 (062)	18.5 (10)	2.08	0.150	6.6 (075)	16.2 (11)	8.76	0.003
Smoking (yes)	46.0 (243)	46.3 (25)	0.001	0.969	43.3 (489)	42.6 (29)	0.01	0.919
Stressful childhood experiences								
Emotional neglect (yes)	42.6 (225)	59.3 (32)	5.51	0.019	48.9 (553)	58.8 (50)	2.51	0.113
Psychological neglect (yes)	25.2 (133)	40.7 (22)	6.06	0.014	33.3 (376)	29.4 (20)	0.43	0.511
Physical abuse (yes)	15.0 (079)	22.2 (12)	1.96	0.162	18.2 (206)	17.6 (12)	0.02	0.904
Sexual abuse (yes)	08.5 (045)	20.4 (11)	7.91	0.005	27.3 (309)	32.4 (22)	0.80	0.370

1 The differences in percentages and mean scores between patients with an opposite-sex and a same-sex orientation are considered as significant when *P *<* *0.01.

SD, standard deviation.

### Lifestyle factor: Substance use

Substance use (alcohol and drug use, and smoking) was not more or less frequent in men with same-sex attraction compared to heterosexual men (see also Table 1988). For women, we observed that illicit drug use was significantly more often reported by women with same-sex attraction, compared to women with heterosexual attraction (Table 1988); differences in the use of alcohol and smoking were not significant.

### Vulnerability indicator: Stressful childhood experiences

In comparison to men with heterosexual attraction, men with any same-sex attraction more often reported experiences of sexual abuse as a child. There were no sexual attraction-related differences in emotional and psychological neglect and physical abuse (Table 1988). For women there were no significant differences in the childhood trauma variables between those with any same-sex attraction and with heterosexual attraction (see also Table 1988).

### Sensitivity analyses

Results from the chi-square and *t*-tests showed that no associations were found between sexual attraction and the studied clinical expression variables. With regard to illicit drug use and childhood trauma the analyses found that for men there was a significant association between same-sex orientation and sexual abuse during childhood and for women there was an association with illicit drug use. We therefore only assessed with a multiple logistic regression analyses whether the significant differences in men and women were independent of possible demographic confounders, including age, level of education, and having a steady partner.

As showed in the first part of Table 2011, for men sexual abuse still was related to sexual orientation after controlling for age, education level, and having a steady partner. For women illicit drug did also remain significantly associated with sexual attraction after controlling for the demographic variables (age, level of education, and having a partner relation; see Table 2011).

**Table 3:** Results of multivariable logistic regression analyses predicting sexual attraction, separately for men and women

	OR	*P*	95% Confidence interval
Men			
Controlling variables: Social demographics			
Age	01.02	0.166	0.99–001.05
Level of education			
Basic (reference group)			
Intermediate	04.86	0.134	0.61–038.43
High	13.05	0.015	1.66–102.68
Relationship status	00.36	0.001	0.20–000.67
Childhood trauma			
Sexual abuse	03.09	0.00	1.43–006.69
Women			
Controlling variables: Social demographics			
Age	01.00	0.940	0.98–01.02
Level of education			
Basic (reference group)			
Intermediate	04.17	0.164	0.56–31.24
High	13.44	0.011	1.81–99.97
Relationship status	00.82	0.458	0.15–11.37
Life style factor			
Illicit drug use	3.33	0.001	1.61–006.87

OR, odds ratio.

## Discussion

To our knowledge, this is the first study to examine differences in clinical expression, lifestyle factors, and risk indicators for psychopathology between patients diagnosed with a past-year DSM-IV depressive and/or anxiety disorder, who have any same-sex attraction versus heterosexual attraction. The clinical expression of mood and anxiety disorders (including severity of disorder and loneliness) did not seem to differ between patients of both sexes with and without same-sex attraction. However, we found a substantial difference for women regarding illicit drug use, and for men regarding sexual abuse during childhood.

It should be noted that in the present study, carried out in the Netherlands, 6.9% (*n *=* *122; 54 men and 68 women) of the patients with a past-year depressive and/or anxiety disorder reported to experience same-sex attraction. This percentage is higher than that found in the Netherlands Mental Health Survey and Incidence Studies (NEMESIS) which is a longitudinal study focusing on the incidence, prevalence, course, and consequences of mental health problems in the general population between 18–64 years old in the Netherlands. In NEMESIS II (data collected in 2007–2009), for example, 2.2% could be identified as a sexual minority (Gevonden et al. 2014). In contrast to our study, NEMESIS surveys a general population sample and does not only include participants who have a diagnosis with a past-year DSM-IV depressive and/or anxiety disorder.

The higher percentage of sexual minorities in our patient sample compared to national representative samples is in line with studies that showed that lesbian, gay, and bisexual (LGB) people report more lifetime depressive and anxiety disorders (Sandfort et al. 2001; Bostwick et al. 2010) and that LGB persons seeking more frequently professional help for their problems (Bos et al. 1999; Rothblum and Factor 2001; King et al. 2003; Chakrabortly et al. 2011). Numerous scholars have emphasized that the institutional and interpersonal discrimination that sexual minorities experience might be an important explanation for the mental health disparities between LGB and heterosexual persons (Mays and Cochran 2001; Hatzenbuehler et al. 2009).

Also, the relatively high percentage (i.e., 5.7%) of female patients in our study with attraction to both sexes is in line with national population-based studies. Bostwick et al. (2010) used data from a national population study in the United States and found among bisexual women the highest rates of DSM-IV mood and anxiety disorders compared to heterosexual women but also compared to lesbian women (Bostwick et al. 2010).

The absence of significant differences in clinical expression between patients with and without same-sex attraction might be explained by the relatively higher educational level among persons with same-sex attraction. Educational level might have a protective impact on the association between sexual orientation and clinical expression in this sample of patients. Another explanation for the absence of significant differences in clinical expression might have to do with the way sexual orientation was measured. It might be that when sexual orientation was measured differently (for example, by asking about sexual behavior and/or sexual identity) our findings would have been different. It might furthermore matter whether patients with same-sex attraction had disclosed their feelings to others. Those who are not open might suffer more and therefore might show more severe clinical expression. There is evidence in a nonclinical sample, for example, that adult LGB persons who have disclosed their sexual identity showed lower levels of cortisol and reported less psychiatric symptoms, compared to those we did not disclose (Juster et al. 2013).

Regarding lifestyle factors we found that compared to heterosexual women, those with any same-sex attraction more often reported illicit drug use during the last month. The literature already has documented a higher risk for substance use in sexual minority women compared with heterosexual women (Burgard et al. 2005; Hughes et al. 2010). It is not clear whether this substance use is related to experiences with stigmatization or that it is part of an unhealthy lifestyle related to being a part of a lesbian community.

For the vulnerability factors we found that experiences with sexual abuse during childhood was the only childhood trauma factor on which we found a significant difference between male patients with same-sex attraction compared to heterosexual attraction. Studies in nonpatient populations also showed that compared to heterosexual men, gay and bisexual men are more likely to report childhood sexual abuse (Corliss et al. 2002; Balsam et al. 2005; Friedman et al. 2011). As such, the differences in childhood trauma between same-sex attracted and heterosexual male patients that were found in the present study might reflect a different etiology of mental disorders.

In the multiple logistic regression analyses in which we controlled for age, educational level, and having a steady partner we found the same results, indicating that women and men with any same-sex attraction were more likely to report illicit drug use and sexual abuse during childhood, compared to heterosexual women and men, respectively.

Several limitations should be taken into account. First, no information was gathered about the concealment of one’s same-sex attraction and therefore the association between nondisclosure and clinical expression and the risk indicator for depressive and/or anxiety disorders could not be tested. Second, the numbers were too small to look at differences between bisexual patients and those who exclusively have same-sex attraction. Third, the study is cross-sectional and therefore no conclusions can be drawn about the direction of the associations between sexual attraction and clinical expression and the risk indicators for depressive and/or anxiety disorders.

## Conclusion

Although we found no associations between sexual attraction and clinical expression, our findings point at differences in lifestyle factors (for women) and vulnerability indicators (for men) in depressed and/or anxious patients. For female patients with any same-sex attraction we found higher rates of drug use, which might reflect coping mechanisms for dealing with existing symptoms or with minority stressors they have to deal with. For men, childhood trauma might be an important factor in the etiology of depressive and/or anxiety disorders in male patients with same-sex attraction.

The results of this study highlight the need to give appropriate attention to substance use and childhood trauma in treatment plans for same-sex attracted female and male patients with depression and/or anxiety disorders.
